# The Innovative and Sustainable Use of Dental Panoramic Radiographs for the Detection of Osteoporosis

**DOI:** 10.3390/ijerph17072449

**Published:** 2020-04-03

**Authors:** Andy Wai Kan Yeung, Ioana Mozos

**Affiliations:** 1 Oral and Maxillofacial Radiology, Applied Oral Sciences and Community Dental Care, Faculty of Dentistry, The University of Hong Kong, Hong Kong, China; 2 Department of Functional Sciences, “Victor Babes” University of Medicine and Pharmacy, 300173 Timisoara, Romania; 3 Center for Translational Research and Systems Medicine, “Victor Babes” University of Medicine and Pharmacy, 300173 Timisoara, Romania

**Keywords:** panoramic radiograph, osteoporosis, dental radiology, computer-aided diagnosis, literature analysis, digital dentistry

## Abstract

This bibliometric study evaluated the scientific impact of papers dealing with osteoporosis detected by dental panoramic radiographs by performing citation analysis and cited reference analysis. Retrospective data was extracted from the Web of Science Core Collection database and imported into VOSviewer, CRExplorer, and CitNetExplorer for analyzing semantic contents, cited references, and temporal citation network. The 280 relevant papers identified were cited 4874 times, having an h-index of 38 and 17.4 citations per paper. The top five major contributing countries were Japan (*n* = 54, 19.3%), USA (*n* = 43, 15.4%), Brazil (*n* = 38, 13.6%), Turkey (*n* = 38, 13.6%), and the UK (*n* = 32, 11.4%). Citation per paper correlated with publication count among the authors and institutions. Mandibular cortical width was the most frequently used and most cited measurement index. References published during the 1970s and 1980s have built the foundation for the development of research that investigates the potential associations between osteoporosis and radiographic measurements on panoramic radiographs. Osteoporosis detection by dental panoramic radiographs is a perennially investigated research topic with global contributions. Panoramic radiographs are considered early detection and screening tools for osteoporosis by worldwide research.

## 1. Introduction

Analyzing bibliometric data helps us understand research trends and hot topics, which are also important and getting attention in the medical fields [1,2]. It was estimated that more than 49 million people around the world are affected by osteoporosis, based on a 2014 study [3]. Osteoporosis is a generalized condition which also affects the alveolar bone [4]. As medical advancement has prolonged the general life expectancy, the prevalence of osteoporosis will probably increase. To diagnose osteoporosis by determining the bone mineral density, the dual-energy X-ray absorptiometry (DXA) is one of the most reliable modalities [5]. Though it is non-invasive, it is not readily available in many countries. For instance, a survey has reported that there were only 3000 patients being screened by DXA in each of the 23 European centers that responded [6]. There are other biomarkers in osteoporosis obtainable in an invasive manner, such as oxidative stress markers in blood samples [7,8]. On the other hand, dental panoramic radiography is cheaper and more readily available from dental clinics and hospitals and is equipped in the less developed countries as well [9]. Panoramic radiographs are frequently used during routine dental checkups and before common dental treatment procedures [10]. Though panoramic radiography is not a recognized diagnostic tool for osteoporosis, its utilization in detecting or screening for osteoporosis might potentially benefit a larger number of patients, including those primarily seeking dental treatments [11]. It has been repeatedly demonstrated that the measurements on panoramic radiographs may identify or help detect osteoporosis [12,13,14,15]. One relevant question remained unanswered: Are these studies being read by academics around the world so that they can transform the evidence-based clinical practice? A bibliometric study can identify and assess the relevance of academic publications by analyzing their citation performance and selection of cited references, hence enabling a better understanding of the overall research landscape of the research field [16,17,18]. Instead of a qualitative evaluation of the existing evidence by means of a meta-analysis [19,20,21], a bibliometric study can qualitatively evaluate the influence of the predefined literature and identify the seminal works within it. There have been abundant bibliometric studies on numerous aspects of radiology [22,23,24]. However, none of them pertains to the innovative use of dental panoramic radiographs to detect osteoporosis that affects the whole body.

Therefore, the primary aims of this study are to reveal the overall citation performance of papers dealing with osteoporosis detection by panoramic radiographs and identify the major contributors, the most cited papers, and the important references and hot topics. The secondary aim is to reveal the main indices used for mandibular bone measurements as early detection and screening tools for osteoporosis.

## 2. Materials and Methods

Retrospective data was extracted from the Web of Science (WoS) database hosted by Clarivate Analytics. IRB or ethical approval was not required for this study.

The search strategy was defined as Topic=(osteoporosis) AND Topic=(panoramic). The search included papers with both the words osteoporosis and panoramic in the title, abstract or keywords. Only articles and reviews were considered. The background data of the papers were analyzed descriptively, which included author, country, institution, language, journal, and publication year. The top 10 most cited papers were identified.

We downloaded the full records of the papers yielded from the search and imported into a bibliometric software called VOSviewer [25] for further citation analyses. The processing pipeline and algorithms used by VOSviewer have been previously documented [26,27]. It was utilized in the current study to visualize a bubble map that analyzed the terms (i.e., words or phrases) from titles and abstracts of the analyzed papers. For simplicity, only those appeared in at least ten of the included papers were further processed. General and irrelevant noun phrases were removed via an algorithm [28] and by manual inspection of the bubble map initially generated [17,29]. The bubble size indicates the number of papers containing the term. The bubble color indicates its citation per paper. Terms that co-appear frequently in the same papers have their bubbles positioned closer to each other.

Cited references of all included papers were analyzed by CRExplorer [30], which identifies the historical roots of the research field by plotting a “reference publication year spectroscopy” (RPYS). RPYS illustrates the changes in the frequency with which the references were cited by considering the publication years of the cited references. Briefly, RPYS reveals in which years the references published were more highly cited than the two preceding and two succeeding years. These references might not have directly investigated osteoporosis detection by panoramic radiographs, but they acted as the cornerstones for the subsequent studies. To further examine how these cited references influenced the subsequent studies, CitNetExplorer [31] was used to visualize a temporal citation network among the 100 most cited references.

We first performed descriptive data analysis. Analytical statistics, namely Pearson’s correlation tests, were subsequently performed to analyze whether there existed a significant correlation between citation per paper and publication count with regard to authors, institutions, countries, and journals. Data were analyzed with the Statistical Package for Social Sciences (SPSS version 24.0, SPSS Inc, Chicago, IL, USA). Test results were statistically significant if *p* < 0.05.

## 3. Results

The search in WoS yielded 280 papers: 263 of which were articles and 17 were reviews. They were published in 113 journals from 1991 to 2018 by 969 authors with 312 affiliations from 42 countries. Most of the papers were published in English (*n* = 274, 97.9%). The 280 papers were cited 4874 times in total, having an h-index of 38 and 17.4 citations per paper.

### 3.1. Major Contributors

The top five most prolific authors were from Hiroshima University (Professor Taguchi has since moved to Matsumoto University) and the University of Manchester, and, thus, these two institutions were among the most prolific research bases (Table 1). Brazil was the third most prolific country behind Japan and the USA, and the University of São Paulo was one of the top five most prolific institutions. However, it seemed that Brazilian contributions, in general, had fewer citations per paper relative to the contributions from the USA, UK, and Japan. The top five journals have published 37.5% of the 280 papers. *Osteoporosis International* was the only non-dental journal among the top five, with the second-best citations per paper (33.1) among the five. When examining all contributors, publication count and citation per paper had a significant correlation for authors (r = 0.115, *p* < 0.001) and institutions (r = 0.130, *p* = 0.025) but not for countries (r = 0.140, *p* = 0.371) and journals (r = 0.153, *p* = 0.105).

### 3.2. Most Cited Papers

The top 10 most cited papers are listed in Table 2. They were published between 1991 and 2002. Each of them had 87–169 citations. Six of them had more than 100 citations. Relating these 10 papers to the top five most prolific authors listed in Table 1, Taguchi has contributed to three of these 10 papers, whereas Devlin and Horner each had one, and Tanimoto and Suei each had two.

### 3.3. Bubble Map

**Figure 1:**
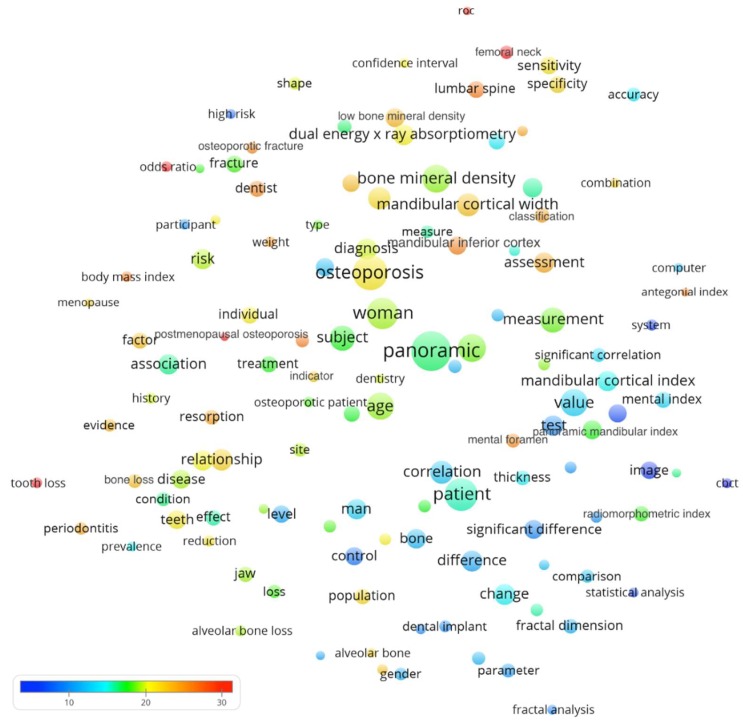
Bubble map showing words or phrases that appeared in at least ten of the 280 papers. We excluded generic terms by a visual inspection of the map generated in the preliminary stage. After screening, 123 terms remained. The bubble size indicates the number of papers containing the term. The bubble color indicates its citation per paper. If the terms frequently co-appeared in the same papers, their bubbles were in closer proximity. The *x*- and *y*-axis carried no specific information and only proximity between bubbles should be considered. Some of the measurement indices might appear under different names.

From Figure 1, one could observe several terms with a huge number of citations per paper (as indicated by red or orange bubbles) include tooth loss (33.9 citations per paper, CPP), postmenopausal osteoporosis (33.4), femoral neck (31.8), odds ratio (31.5), and ROC curve (roc, 29.2). Regarding terms of measurement indices from panoramic radiographs, the mandibular cortical width appeared in 75 papers and had 22.4 citations per paper, followed by mandibular cortical index (56; CPP = 15.1), panoramic mandibular index (50; CPP = 17.6), radiomorphometric index (32; CPP = 17.8), and antegonial index (10; CPP = 25.2). From the bubble map, it seemed that periodontitis (and its related tooth loss) was an important topic involved in the research.

### 3.4. Reference Publication Year Spectroscopy (RPYS)

**Figure 2:**
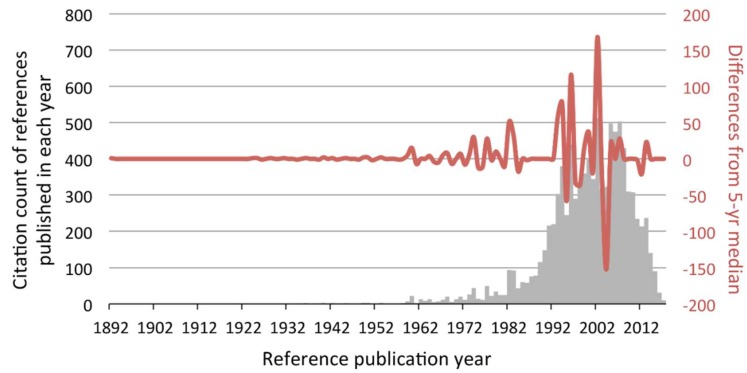
Reference publication year spectroscopy (RPYS) based on the references made by the 280 papers. Each bar (grey) represented the total citation count received by all references published in that particular year. The line (red) shows the difference in the annual citation count from its 5-year median. Take the largest positive peak (year 2002) as an example. References published in years 2000, 2001, 2002, 2003, and 2004 received 397, 344, 512, 318, and 323 citations, respectively. The 5-year median was 344. As references published in year 2002 received 512 citations, the difference was +168 and hence formed a large positive peak.

As said, the first of the 280 papers were published in 1991. When their references were analyzed, the largest positive peaks shown in the RPYS before 1991 were 1974, 1977, and 1982–1983 (Figure 2). The respective representative references published in those years were listed in Table 3. There was one dominant reference in 1974 and 1977 and two in 1982 and 1983, respectively, making a total of six representative references. Five of them were published in dental journals and the remaining one in a statistics journal.

### 3.5. Temporal Citation Network of the Important References

**Figure 3:**
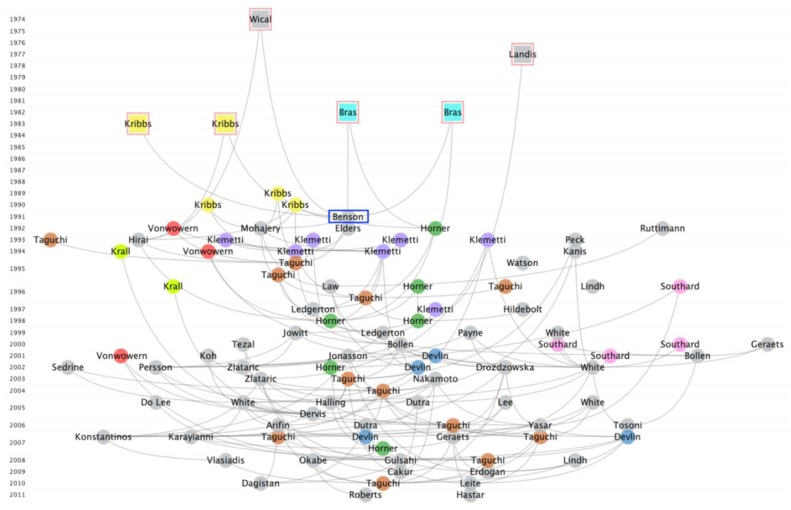
Temporal citation network of the 100 most cited references. The visualization has confirmed that the six representative references (red squared) identified by reference publication year spectroscopy (RPYS) were among the earliest most cited references. They have been similarly cited by many subsequent references, forming a complex intertwined network. For simplicity, transitive reduction was applied, meaning that a citation link between two references would not be shown if they were also connected via citation links with intermediate references visualized in the network. It could be observed that Kribbs has published five important references as the first author for the 280 papers on osteoporosis detection by panoramic radiographs. Note that the first of the 280 papers was published in 1991 by Benson et al. (Ranked 4th in Table 1), which was naturally a highly cited reference by itself (blue squared). The horizontal location of a publication is determined by its citations relations with other publications. The author who had several articles was marked with the same color. Due to the limitation of the software, only 9 authors could be colored.

To better visualize how the abovementioned six representative references have influenced the subsequent references, a temporal citation network was synthesized. They were the earliest ones among the 100 most cited references by the 280 papers (Figure 3). Apparently, all these six representative references were cited by many of the subsequent works that formed a complex intertwined citation network. Together with more works published by Kribbs and co-workers in 1989 and 1990, they laid the foundation for the research on osteoporosis detection by panoramic radiograph.

## 4. Discussion

This bibliometric study has affirmed that papers on osteoporosis detection by panoramic radiographs have received a large number of citations per paper (17.4). The analyses mainly concerned top-cited papers. The correlational results were consistent with the stated hypothesis for the authors and institutions but not for the countries and journals. The topic has received global contributions from Asia, the United States, Europe, and South America. Notably, Japan was the most prolific country on this topic, despite the fact that the United States has been the traditional outstanding dominating power in terms of publishing scientific papers such as in the fields of osteoporosis [32] and radiology [33]. Japanese researchers obviously had large inputs into this research topic. For example, Professor Taguchi and co-workers have demonstrated the reliability of mandibular cortical width and morphology in diagnosing postmenopausal osteoporosis [13]. Following that, they and Professor Katsumata have identified factors affecting inter-observer agreement on such evaluations [34] and hence the development of computerized measurements by software [35]. From Table 1 and Table 2, it can be observed that most of the highly cited papers and their representative references were published in either *Osteoporosis International* or specialized oral and maxillofacial radiology journals within dentistry. It demonstrates that most of the relevant literature were published in specialized journals. The earliest work in Table 2 was by Benson et al. in 1991, which reported a significant reduction in panoramic mandibular index upon aging among women of certain racial groups, but vice versa in white men [36]. Three years later, Klemetti et al. in Finland published the work ranked 1st in Table 2, which reported an association between panoramic measurement indices and skeletal mineral status, but a low sensitivity of diagnosing osteoporosis risk [37]. These early findings did not discourage researchers from conducting follow up studies. A case–control study (ranked 5th) observed 2–8 times of increased risk of self-reported osteoporotic fracture from patients with moderate to severe thinning of the mandibular lower cortex [38]. Meanwhile, researchers from the UK established a diagnostic threshold for mandibular cortical width (mental index) of 3 mm or less as an appropriate criterion to refer for bone densitometry (ranked 3rd) [39].

From Table 1 and Figure 2, it seemed that periodontitis was an important topic in the investigation of osteoporosis. Indeed, it was suggested that there were mixed pieces of evidence on the relationship between osteoporosis and periodontitis, in which the potential confounding factors and variety of assessment methods have made it even more complicated [40]. Klemetti et al. have found that, with regard to the ability to retain their teeth with severe periodontitis, individuals with higher bone mineral values seemed to perform better than those with osteoporosis [41], implying that osteoporotic patients might lose their teeth more easily. Meanwhile, it was reported that osteoporosis and mandibular cortical index were associated with horizontal alveolar bone loss (periodontitis) [42]. Besides, the reduction in mandibular bone mass was positively correlated with tooth loss among women [43]. All these findings still could not clarify the causal relationship between osteoporosis and periodontitis. The link between osteoporosis and periodontitis is probably represented by inflammatory cytokines, such as interleukin-1, -6 and tumor necrosis factor alpha, responsible for bone loss in osteoporosis due to their effects on osteoclast activity and destruction of tooth-supporting tissues [4]. Both periodontitis and osteoporosis shared multiple risk factors, including age, genetics, hormonal disruptions, smoking, and deficiency of calcium and vitamin D [44]. The presence of osteoporosis was found to increase the risk of having periodontitis by 22% [45]. Meanwhile, the latest study reported that antiosteoporosis medication directly predicted both periodontitis and systemic bone status, but the severity of periodontitis did not associate with bone mineral density changes [46]. This would certainly be a popular topic in future studies.

A previous meta-analysis published by Calciolari et al. [19] identified three indices that were mostly used for mandibular bone measurements: the mandibular cortical width (also known as mandibular cortical thickness, or mental index), the panoramic mandibular index, and the mandibular cortical index (also known as the Klemetti index). Mandibular cortical width is measured at the mental foramen region, along a vertical line joining the mental foramen and a tangential line from the inferior border of the mandible. Panoramic mandibular index calculates the ratio between the mandibular cortical width at the mental foramen area and the distance between the inferior edge of the foramen and the inferior border of the mandible. Meanwhile, the mandibular cortical index morphologically classifies the cortex distal to the mental foramen into three categories. The meta-analysis concluded that the mandibular cortical width had the best specificity, whereas all three indices had variable sensitivity reported by different studies [19]. In the current study, mandibular cortical width was found to have the highest citations per paper among the three indices (7.3 more citations per paper than the mandibular cortical index, and 4.8 more citations per paper than the panoramic mandibular index). It was also the most frequently used index, among the three, in the 280 papers. Combined together, these findings suggested that mandibular cortical width was one of the indices receiving the most attention and the best performance and should continue to be used as a potent marker during evaluation. It should be noted that some of the measurement indices might appear under different names in the papers, and, thus, the results could be even higher if the alternative names were considered together.

In the current study, six important references were published before 1991, when the first of the 280 osteoporosis papers analyzed were published. One of these six references was a statistics paper that presented a methodology of using kappa statistics to test inter-observer agreement [47]. This surely is very important for papers reporting measurements from multiple observers. The work by Wical and Swoope in 1974 reported the spatial relationship between mental foramen and inferior border of the mandible remained stable regardless of alveolar ridge resorption [48]. Though it was intended to be published with a prosthodontics implication for teeth replacement, subsequent studies could apply their findings to devise mandibular measurement indices. Meanwhile, the works by Bras et al. in 1982 reported that postmenopausal women over 60 years old or patients with chronic renal failure had distinctly thinner angular cortex at gonion of the mandible, as measured from panoramic radiographs [49,50]. These patient groups were not directly osteoporotic but could still inspire following osteoporosis studies to apply mandibular measurements to detect such bone loss. Last but not least, the works by Kribbs et al. in 1983 have measured bone loss in the inferior border of the mandible by imaging “enlarged periapical radiographs” by occlusal films and evaluating them with microdensitometry [51,52]. These earlier seminal works have provided the foundation for subsequent studies that focused on osteoporosis detection by panoramic radiographs.

The current study has several limitations. Firstly, relevant papers not indexed in WoS, preprints uploaded to servers such as arXiv and bioRxiv, and unpublished papers were not identified and included in the current analysis. However, WoS is often considered as the “gold standard” for extracting bibliometric data for analysis because it is more accurate and better at tracking data from older publications [53]. Meanwhile, the interpretation of citations may not be straightforward, as it was not possible to determine if the citations were inaccurate, negative (e.g., questioning or rebutting claims from cited references), or selective (e.g., intentionally omitting some relevant references). For instance, there might exist a “snowball effect” in the citation behavior, meaning that there is a tendency to cite papers or authors that are already highly cited [54]. The validity or usefulness of panoramic radiographs was not directly assessed in the current study, which depends on various factors, such as the different experiences of operators, image quality, and image magnification [19].

## 5. Conclusions

Within the constraints of the limitations of the study, the following can be concluded:

- On average, papers dealing with osteoporosis detection by panoramic radiographs have received 17.4 citations per paper.
- These papers have received global contributions from Asia, the United States, Europe, and South America, with Japan and Brazil among the largest contributors.
- Most of the papers and references were published in journals specialized in osteoporosis or oral and maxillofacial radiology.
- Periodontitis was one of the topics with many citations per paper.
- Mandibular cortical width was the most frequently used and most cited measurement index relative to mandibular cortical index and panoramic mandibular index.
- Important references were published during the 1970s and 1980s that have built the foundation for the development of research that investigates the potential associations between osteoporosis and radiographic measurements on panoramic radiographs.
- Though, currently, panoramic radiograph is not a recognized tool for diagnosing osteoporosis, its reliability in screening / detecting osteoporotic patients has been frequently investigated and cited.

## Figures and Tables

**Table 1:** The top 5 major contributors in terms of author, country, institution, and journal.

Contributor	Number of Papers (%)	Citations per Paper
*Author*		
Taguchi, Akira	31 (11.1%)	37.2
Devlin, Hugh	29 (10.4%)	34.1
Horner, Keith	24 (8.6%)	38.1
Tanimoto, Keiji	17 (6.1%)	51.3
Suei, Yoshikazu	16 (5.7%)	51.9
*Country*		
Japan	54 (19.3%)	20.3
USA	43 (15.4%)	30.4
Brazil	38 (13.6%)	11.1
Turkey	38 (13.6%)	7.8
UK	32 (11.4%)	32.3
*Institution*		
University of Manchester	30 (10.7%)	34.6
Hiroshima University	24 (8.6%)	38.9
University of São Paulo	12 (5.0%)	4.9
KU Leuven	12 (4.3%)	30.9
Academic Center for Dentistry Amsterdam ^1^	10 (3.6%)	35.0
*Journal*		
Dentomaxillofacial Radiology	42 (15.0%)	23.2
Oral Surgery, Oral Medicine, Oral Pathology, and Oral Radiology ^2^	29 (10.4%)	33.7
Osteoporosis International	15 (5.4%)	33.1
Clinical Oral Investigations	11 (3.9%)	5.3
Oral Radiology	8 (2.9%)	2.9

^1^ Institutions with 10 papers equally were Asahi University, Matsumoto Dental University, and the University of Athens. ^2^ This journal has been renamed; papers published in its predecessors were counted under the same entity.

**Table 2:** Top ten most cited papers.

Rank	Paper	Total Citations
1	Klemetti E, Kolmakov S, and Kröger H., Pantomography in assessment of the osteoporosis risk group. *Eur J Oral Sci* 1994,102:68–72.	169
2	Wactawski-Wende J, Grossi SG, Trevisan M, et al. The role of osteopenia in oral bone loss and periodontal disease. *J Periodontol* 1996,67:1076–1084.	132
3	Devlin H. and Horner K., Mandibular radiomorphometric indices in the diagnosis of reduced skeletal bone mineral density. *Osteoporos Int* 2002,13: 373–378.	110
4	Benson BW, Prihoda TJ, Glass BJ. Variations in adult cortical bone mass as measured by a panoramic mandibular index. *Oral Surg Oral Med Oral Pathol* 1991,71:349–356.	105
5	Bollen AM, Taguchi A, Hujoel PP, et al. Case–control study on self-reported osteoporotic fractures and mandibular cortical bone. *Oral Surg Oral Med Oral Pathol Oral Radiol Endod* 2000,90: 518–524.	102
6	Taguchi A, Suei Y, Ohtsuka M, et al. Usefulness of panoramic radiography in the diagnosis of postmenopausal osteoporosis in women. Width and morphology of inferior cortex of the mandible. *Dentomaxillofac Radiol* 1996,25:263–267.	100
7	Klemetti E, Collin HL, Forss H, et al. Mineral status of skeleton and advanced periodontal disease. *J Clin Periodontol* 1994,21:184–188.	93
8	Hirai T, Ishijima T, Hashikawa Y, et al. Osteoporosis and reduction of residual ridge in edentulous patients. *J Prosthet Dent* 1993,69:49–56.	92
9	Mohajery M, Brooks SL. Oral radiographs in the detection of early signs of osteoporosis. *Oral Surg Oral Med Oral Pathol* 1992,*73*:112–117.	90
10	Taguchi A, Tanimoto K, Suei Y, et al. (1995). Tooth loss and mandibular osteopenia. *Oral Surg Oral Med Oral Pathol Oral Radiol Endod* 1995,*79*:127–132.	87

**Table 3:** Representative references from positive peaks (years 1974, 1977, 1982–1983) of reference publication year spectroscopy (RPYS).

Year	Paper	Total Citations from the 280 Papers
1974	Wical KE, Swoope CC. Studies of residual ridge resorption. Part I., Use of panoramic radiographs for evaluation and classification of mandibular resorption. *J Prosthet Dent* 1974,*32*:7–12.	22
1977	Landis JR, Koch GG. The measurement of observer agreement for categorical data. *Biometrics* 1977,33:159–174.	17
1982	Bras J, Van Ooij CP, Abraham-Inpijn L, et al. Radiographic interpretation of the mandibular angular cortex: A diagnostic tool in metabolic bone loss: Part I., Normal state. *Oral Surg Oral Med Oral Pathol* 1982,*53*:541–545.	41
	Bras J, Van Ooij CP, Abraham-Inpijn L, et al. Radiographic interpretation of the mandibular angular cortex: A diagnostic tool in metabolic bone loss: Part II. Renal osteodystrophy. *Oral Surg Oral Med Oral Pathol* 1982,*53*: 647–650.	16
1983	Kribbs PJ, Smith DE, Chesnut CH. Oral findings in osteoporosis. Part I: Measurement of mandibular bone density. *J Prosthet Dent* 1983,*50*:576–579.	16
	Kribbs PJ, Smith DE, Chesnut CH. Oral findings in osteoporosis. Part II: Relationship between residual ridge and alveolar bone resorption and generalized skeletal osteopenia. *J Prosthet Dent* 1983,*50*:719–724.	17
